# *One-Stop Shop* para Imagens Cardiovasculares Não Invasivas?

**DOI:** 10.36660/abc.20210245

**Published:** 2021-06-08

**Authors:** Rodrigo Julio Cerci, Afonso Akio Shiosaki

**Affiliations:** 1 Quanta Diagnóstico por Imagem CuritibaPR Brasil Quanta Diagnóstico por Imagem , Curitiba , PR - Brasil; 2 Centro Diagnóstico Hospital Paraná MaringáPR Brasil Centro Diagnóstico do Hospital Paraná , Maringá , PR - Brasil; 3 Ômega Diagnóstico LondrinaPR Brasil Ômega Diagnóstico , Londrina , PR - Brasil

**Keywords:** Tomografia Coronariana, Doença Arterial Coronariana, Perfusão Miocárdica, Doenças Cardiovasculares/diagnostico por imagem, Diagnóstico por Imagem/tendências

Nos últimos quinze anos, a angiotomografia coronariana (ATC) testemunhou rápidos avanços tecnológicos e científicos na detecção de doença arterial coronariana (DAC) anatômica, levando a uma melhora no atendimento ao paciente. ^1^ A avaliação visual da gravidade da estenose pela ATC tem alta sensibilidade e valor preditivo negativo quando comparada à angiografia invasiva, tornando-se um teste ideal para descartar DAC obstrutiva. ^2^ Com seu alto desempenho diagnóstico associado a um importante impacto prognóstico no tratamento da DAC, a ATC finalmente se estabeleceu como uma recomendação de Classe I em diretrizes internacionais ( *European Society of Cardiology* – ESC). ^3^

No entanto, a ATC é limitada por modesta especificidade diagnóstica e fornece apenas avaliação anatômica, o que não informa a significância hemodinâmica de lesões específicas. ^4^ A ATC aliada à avaliação da perfusão miocárdica (CTP) por tomografia de estresse é uma modalidade precisa para determinar as repercussões do fluxo miocárdico regional na estenose coronariana, embora geralmente requeira aquisição adicional e ainda seja subutilizada. ^5^ A reserva de fluxo fracionada por tomografia computadorizada (FFR-CT) é outra abordagem “fisiológica” de TC em que a dinâmica de fluidos computacional é aplicada a dados de ATC padrão e surgiu como uma ferramenta promissora para a avaliação funcional da estenose coronariana. O valor diagnóstico do FFR-CT realizado remotamente foi validado prospectivamente em vários grandes estudos multicêntricos, mas requer o uso de supercomputadores externos, o que pode ser demorado e caro, limitando sua ampla utilidade clínica. ^6 - 8^

O artigo de Morais et al. ^9^ apresentou dados de 93 pacientes submetidos à ATC em scanners de diferentes gerações, aplicando uma técnica FFR-CT que pode ser realizada no local e em tempo real, utilizando ferramentas de inteligência artificial em um *software* protótipo que roda em um estação de trabalho padrão. Essa ferramenta abrevia a necessidade de supercomputadores para realizar cálculos de reserva de fluxo coronariano que geralmente levam até 48 horas, juntamente com um custo adicional para a análise funcional coronariana que atualmente é realizada por um *software* externo exclusivo, impedindo o acesso universal a todos os pacientes que poderiam se beneficiar com essa tecnologia. Ao contrário do FFR-CT externo, o FFR-CT interno estima a reserva de fluxo coronário por um algoritmo de aprendizado profundo baseado em mapas anatômicos das artérias coronárias, bem como graus de estenose. ^10^

Embora limitado pelo viés de referência de uma análise relativamente pequena, unicêntrica e retrospectiva, os autores devem ser parabenizados por reproduzir resultados semelhantes quando comparados a estudos maiores de FFR-CT externo. Isso significa que se podem esperar os mesmos resultados, bem como as mesmas limitações para o FFR-CT no local. Deve-se notar que os dados são consistentes com os achados de vários estudos nos quais, comparados à ATC e ao SPECT, a FFR-CT tem acurácia diagnóstica superior na discriminação de isquemia (AUC = 0,93). ^6 , 7 , 11 - 13^

Para aplicação de rotina, no entanto, os médicos devem ter em mente que o ponto de corte da FFR-CT de <0,80 derivou uma taxa de falsos negativos de 12%, enquanto um ponto de corte de <0,85 derivou apenas 6% de falsos negativos e pode ser mais conservador e de abordagem mais segura para o uso da FFR-CT como porta para uma angiografia invasiva.

Infelizmente, a FFR-CT não é para todos os pacientes, pois a avaliação da patência do *stent* ou do enxerto ainda não foi validada. Além disso, lesões pesadas calcificadas, ostiais e bifurcadas permanecem um desafio. Outro obstáculo importante é a qualidade da imagem, que precisa estar livre de artefatos de movimento e para ser processada, deixando uma taxa de rejeição variável, mas significativa, de 3 a 20%. ^13 , 14^

No entanto, a possibilidade de uma FFR-CT no local tem sido o sonho dos imageadores cardiovasculares, integrando dados anatômicos e fisiológicos em um único conjunto de dados de aquisição ( *one-stop shop* ), aumentando a resolução do teste de forma democrática, com muito menos tempo de análise e custos em comparação com a FFR-CT externa. O artigo de Morais et al. ^9^ nos aproxima do “sonho que se torna realidade”.
