# Supplementary material for: One-Stop Shop para Imagens Cardiovasculares Não Invasivas?
Source: Arq Bras Cardiol. 2021 Jun 8;116(6):1099–100. [Article in Portuguese] doi: 10.36660/abc.20210245 (PMC8288530; doi:10.36660/abc.20210245)
Supplement: Translation

# Translation: One-Stop Shop for Non-Invasive Cardiovascular Imagers?

**Authors:** Rodrigo Julio Cerci, Afonso Akio Shiosaki

**Affiliations:** 1 Brasil Quanta Diagnóstico por Imagem, Curitiba, PR - Brasil; 2 Brasil Centro Diagnóstico do Hospital Paraná, Maringá, PR - Brasil; 3 Brasil Ômega Diagnóstico, Londrina, PR - Brasil

**Keywords:** Coronary, Tomography, Coronary Artery Disease, Myocardial Perfusion, Cardiovascular Diseases/diagnostic, imaging, Diagnostic, Imaging/trends

Over the past fifteen years, coronary computed tomography angiography (CCTA) has witnessed rapid technological and scientific advances in the detection of anatomical coronary artery disease (CAD), leading to an improvement in patient care.^1^ Visual assessment of stenosis severity using CCTA has a high sensitivity and negative predictive value when compared to invasive angiography, making it an ideal test to exclude obstructive CAD.^2^ With its high diagnostic performance associated with an important prognostic impact in the management of CAD, CCTA has finally established itself as a Class I recommendation in international guidelines (European Society of Cardiology – ESC).^3^

However, CCTA is limited by modest diagnostic specificity and only provides anatomical assessment, which does not inform hemodynamic significance of specific lesions.^4^ CCTA combined with stress tomography evaluation of myocardial perfusion (CTP) is an accurate modality to determine regional myocardial flow repercussions of coronary stenosis, though it usually requires additional acquisition and is still underused.^5^ Derived flow fractional reserve – computed tomography (FFR-CT) is another “physiologic” CT approach in which computational fluid dynamics is applied to standard CCTA data and has emerged as a promising tool for the functional assessment of coronary stenosis. The diagnostic value of remotely performed FFR-CT has been prospectively validated in several large multicenter studies, but requires the use of offsite supercomputers, which can be time-consuming and cost-intensive, limiting its widespread clinical utility.^6 - 8^

The paper by Morais et al.^9^ presented data from 93 patients submitted to CCTA in scanners from different generations, applying a FFR-CT technique that can be performed on site and in real time, using artificial intelligence tools in a prototype software that runs on a standard workstation. This tool abbreviates the need of supercomputers to perform coronary flow reserve calculations that usually take up to 48 hours, coupled with an additional cost for the coronary functional analysis that is currently performed by unique offsite software, preventing universal access to all patients who could benefit from this technology. Unlike the offsite FFR-CT, onsite FFR-CT estimates the coronary flow reserve by a deep learning algorithm based on anatomical maps of coronary arteries, as well as degrees of stenosis.^10^

Although limited by referral bias from a relatively small, unicenter, and retrospective analysis, the authors must be congratulated for reproducing similar results when compared to larger offsite FFR-CT trials. This means that one may expect the same results, as well as the same limitations for the onsite FFR-CT. It should be noted that the data are consistent with findings of several studies in which, compared to CCTA and SPECT, FFR-CT has superior diagnostic accuracy in discriminating ischemia (AUC = 0,93).^6 , 7 , 11 - 13^

For routine application, however, clinicians must have in mind that the FFR-CT cut point of < 0.80 derived a false negative rate of 12% while a cutoff point of < 0.85 derived only 6% of false negatives and may be a more conservative and safer approach to using FFR-CT as a gatekeeper for invasive angiography.

Unfortunately, FFR-CT is not for all patients, as evaluation of stent or graft patency was not yet validated. Also, heavy calcified, ostial, and bifurcated lesions remain a challenge. Another important hurdle is image quality, which needs to be free of motion and step artifacts to be processed, leaving a variable but significant rejection rate of 3 to 20%.^13 , 14^

Nevertheless, the possibility of an onsite FFR-CT has been the dream of cardiovascular CT imagers, integrating anatomical and physiological data into a single set of acquisition data (one-stop shop), increasing the test’s resolution in a democratic manner, with much less time of analysis and costs when compared to offsite FFR-CT. The article from Morais et al.^9^ brings us closer to the “dream coming true”.
